# The reduction of disability in community-dwelling frail older people: design of a two-arm cluster randomized controlled trial

**DOI:** 10.1186/1471-2458-10-511

**Published:** 2010-08-23

**Authors:** Silke F Metzelthin, Erik van Rossum, Luc P de Witte, Marike RC Hendriks, Gertrudis IJM Kempen

**Affiliations:** 1 Department of Health Care and Nursing Science, School for Public Health and Primary Care (CAPHRI), Faculty of Health, Medicine and Life Sciences, Maastricht University, Maastricht, The Netherlands; 2 Centre of Research on Autonomy and Participation, Zuyd University of Applied Sciences, Heerlen, The Netherlands; 3 Centre of Research on Technology in Care, Zuyd University of Applied Sciences, Heerlen, The Netherlands; 4 Department of Social Medicine, School for Public Health and Primary Care (CAPHRI), Faculty of Health, Medicine and Life Sciences, Maastricht University, Maastricht, The Netherlands; 5 Department of Human Movement Science, Faculty of Health, Medicine and Life Sciences, Maastricht University, Maastricht, The Netherlands

## Abstract

**Background:**

Frailty among older people is related to an increased risk of adverse health outcomes such as acute and chronic diseases, disability and mortality. Although many intervention studies for frail older people have been reported, only a few have shown positive effects regarding disability prevention. This article presents the design of a two-arm cluster randomized controlled trial on the effectiveness, cost-effectiveness and feasibility of a primary care intervention that combines the most promising elements of disability prevention in community-dwelling frail older people.

**Methods/design:**

In this study twelve general practitioner practices were randomly allocated to the intervention group (6 practices) or to the control group (6 practices). Three thousand four hundred ninety-eight screening questionnaires including the Groningen Frailty Indicator (GFI) were sent out to identify frail older people. Based on their GFI score (≥5), 360 participants will be included in the study. The intervention will receive an interdisciplinary primary care intervention. After a comprehensive assessment by a practice nurse and additional assessments by other professionals, if needed, an individual action plan will be defined. The action plan is related to a flexible toolbox of interventions, which will be conducted by an interdisciplinary team. Effects of the intervention, both for the frail older people and their informal caregivers, will be measured after 6, 12 and 24 months using postal questionnaires and telephone interviews. Data for the process evaluation and economic evaluation will be gathered continuously over a 24-month period.

**Discussion:**

The proposed study will provide information about the usefulness of an interdisciplinary primary care intervention. The postal screening procedure was conducted in two cycles between December 2009 and April 2010 and turned out to be a feasible method. The response rate was 79.7%. According to GFI scores 29.3% of the respondents can be considered as frail (GFI ≥ 5). Nearly half of them (48.1%) were willing to participate. The baseline measurements started in January 2010. In February 2010 the first older people were approached by the practice nurse for a comprehensive assessment. Data on the effect, process, and economic evaluation will be available in 2012.

**Trial Registration:**

ISRCTN31954692

## Background

Frailty is highly prevalent in older people; up to 40% of this population is estimated to be frail and an increasing trend is expected [1]. Frailty is related to an increased risk of adverse health outcomes such as acute and chronic diseases, disability and mortality [2-4]. Disability is defined as difficulty or dependency in the execution of the activities of daily living and is associated with increased healthcare utilization and related costs [5]. Next to professional healthcare services, informal caregivers are a source of long-term care for frail older people. However, demographic and social changes such as fewer children, high divorce rates and other care-giving responsibilities reduce the ability of informal caregivers to provide this care [6]. In view of a growing frail older population and restraints in healthcare expenditure and availability of informal care, disability in frail older people is a public health problem [3] and its prevention is considered to be a priority for research and clinical practice in geriatric care [7].

During the last few decades various interventions have been developed targeting frail older people. These show a large diversity in terms of content, the disciplines involved, duration, intensity and setting. Most studies have been conducted in the field of comprehensive geriatric assessment (CGA) or physical exercise [8]. Comprehensive geriatric assessment has been defined as 'a multidimensional, often interdisciplinary, diagnostic process intended to determine a frail older person's medical, psychosocial, and functional capabilities and problems, with the objective of developing an overall plan for treatment and long-term follow-up' [9]. Reviews have shown that the reported effectiveness of CGA studies are inconsistent [8,10-12]. Physical exercise programs for frail older people are mostly effective on frailty components such as physical fitness and balance, but they are less effective on disability outcomes [13]. The use of technology may be effective but more research is needed in this area [14,15]. In conclusion, only a small number of intervention studies have shown beneficial effects with regard to disability prevention and most studies did not report on the long-term effects [13].

A narrative review by Daniëls and colleagues [8] suggested that future community care interventions for frail older people should be directed towards tailor-made, multidisciplinary and multifactorial interventions, with individualized assessment and interventions conducted by a (primary) care team, involving case management and long-term follow-up. These programs may include a physical exercise component for moderately physically frail older people and a technology component tailored to the needs of older people [8]. Other promising elements are techniques for enhancing self management abilities [16-19] and engagement in meaningful social and productive activities, as these foster natural motivation and self-efficacy in older people [20,21].

The present study focuses on a primary care intervention that combines the most promising elements suggested above. This two-arm cluster randomized controlled trial aims to investigate (1) the effectiveness of the intervention with regard to disability (primary outcome) and several secondary functional outcomes, (2) the impact of the intervention on the central informal caregiver with respect to perceived burden on health-related quality of life and (3) the impact of the intervention on healthcare utilization and related costs. In addition, (4) the feasibility of the intervention, including the adherence, will be studied. This article presents the study design and reports on the results of the screening procedure.

## Methods/design

### Study Design

**Figure 1:**
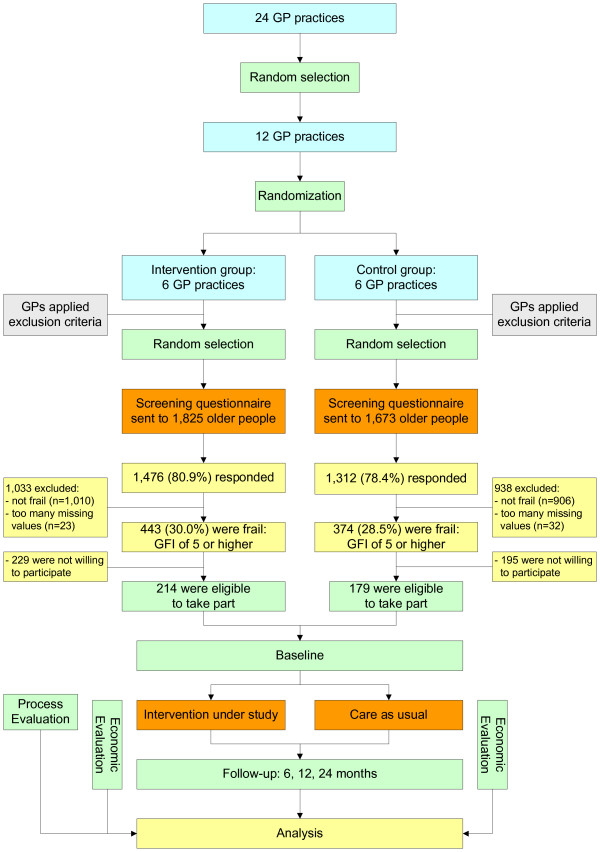
**Study design**. The file contains an overview of the study design.

All general practitioner (GP) practices in the region of Sittard (the Netherlands) and its surroundings were invited to take part in the study, with the restriction that they had no current active and systematic policy for the detection and follow-up of frail older people. In total, 24 GP practices were interested, of which 12 were randomly selected for the study. Cluster randomization was applied to allocate the selected practices to the intervention group (six practices) or the care as usual group (six practices). A flow diagram of the study design is shown in Figure 1.

Effects of the intervention, both for the frail older people and their informal caregivers, will be measured after 6, 12 and 24 months using postal questionnaires and telephone interviews. Data on healthcare utilization and related costs will be gathered continuously over a 24-month period from health insurance registries and registries of GPs and regional hospitals (economic evaluation). A process evaluation will be conducted by means of diaries, questionnaires and semi-structured interviews.

The study obtained approval by the Medical Ethical Committee of the Maastricht University/Academic Hospital Maastricht in the Netherlands in 2009 (MEC 09-3-067).

### Participants and recruitment

The study focuses on community-dwelling frail older people (≥ 70 years). Those who were terminally ill, were confined to bed, had severe cognitive or psychological impairments or were unable to communicate in Dutch were excluded based on the advice of their GP. Consequently, frail older people were screened for frailty. On average, 300 screening questionnaires per practice were sent out depending on the number of older people registered (range 200-350). Where practices had more older people who fulfilled the inclusion criteria, a random selection was drawn. On behalf of their GP, the selected older people received the Groningen Frailty Indicator (GFI) [22] to screen for frailty. In the literature, a score of four or higher (range 0-15) is proposed as the cut-off point for moderately to severely frail older people [22,23]. However, this study focuses on people who are considerably frail. Therefore a cut-off score of 5 was chosen. A letter from the GP, an information leaflet, an informed consent form and a postage free return envelope were included with the screening questionnaire. In total, a sample size of 3,498 older people was addressed (see Figure 1). Reminders were sent to non-responders after three weeks. The selection of participants is performed in two cycles for practical reasons. The first cycle started in December 2009 and the second in February 2010. The undertaking of the intervention and the collection of data will be performed in two cycles as well.

### Randomization

Cluster randomization was applied to avoid contamination bias [24-26]. Before the screening procedure started, six practices were randomly allocated to the new intervention and six practices continued care as usual. Before randomization, the GP practices were pre-stratified into four strata on the basis of practice characteristics, which may influence the results of the study:

1. number of older patients in the practice (< 350 patients versus ≥ 350 patients);

2. urban versus rural area.

It is assumed that GPs working in a practice with a large number of older patients have more experience with geriatric care. Furthermore, it is expected that older people living in a rural area have more support from the informal care system than people living in an urban area.

The practices were stratified in pairs and randomized into either intervention or control group using a computer generated randomization list. To promote extrapolation of the results, practices settled in an urban area with a large number of older people had twice the chance of being allocated to the intervention group than practices in the other three strata.

### Intervention and control group

#### Intervention group

Based on literature studies and an expert meeting a first draft of the intervention protocol was developed by a multidisciplinary task group. This group consisted of a GP, a nursing home physician, a geriatrician, a practice nurse (PN), a home nurse staff member, a nurse specialist, a physical therapist (PT), an occupational therapist (OT), an expert in technology and a researcher as the coordinator. Studies on the screening procedure [27] and on the validity of screening instruments [28] were followed by a pre-pilot study (one GP practice, 10 frail older people) and a pilot study (two GP practices, 50 frail older people) to test the feasibility of the intervention programme (Daniëls et al.: Development of an intervention for community-dwelling frail elderly, submitted). The results were used to develop the final version of the intervention protocol under study.

The GP and the PN are the core team of the intervention with the PN as the case manager. This core team can be extended to include an OT and a PT, or other inpatient and outpatient specialists. The intervention puts emphasis on supporting frail older people to restore or continue the activities they need or enjoy, assuming that participation in social and productive activities is protective against adverse outcomes (Daniëls et al.: Development of an intervention for community-dwelling frail elderly, submitted). The intervention has two main features:

• Identifying risk factors [29] for developing disability and targeting risk factors using professional standards (i.e. Standards of Dutch College of GPs) and the 5A Behavioral Change Model [30].

• Identifying problems in performing activities and enhancing meaningful activities based on the Model of Human Occupation [31].

The intervention takes an individual approach to self-management. The 5A Behavioural Change Model combines a client-centred approach, a model for behavioural change (Stages of Change) and motivational interviewing techniques to provide concrete tools for professionals to support self-management. The 5As refer to the assessment of levels of behaviour, beliefs and motivation, advice adapted to the need for information, the agreement with frail older people on a realistic set of goals, the assistance provided to help them to anticipate barriers and the development of a specific action plan and arrangement of follow-up support [30]. In terms of performing activities, the Model of Human Occupation [31] is considered to be a good tool for analysis and problem-solving. With its base in occupational therapy, the model illuminates how factors of capacity, motivation, lifestyle and environment inter-relate in human occupation. This model has been used in previous successful effect studies [32,33].

**Figure 2:**
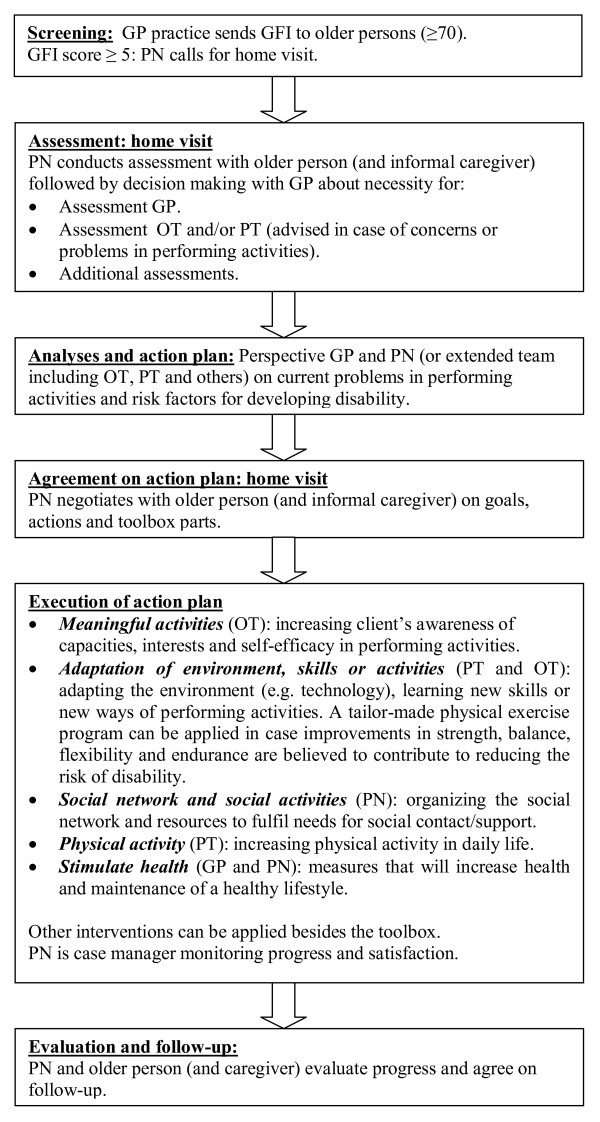
**Steps of the programme**. The file contains an overview of the steps of the programme.

The intervention consists of six steps (see Figure 2). After an initial postal screening (step 1) frail older people (GFI score ≥ 5) will receive a comprehensive multidimensional assessment (step 2) by a PN in collaboration with the GP. This assessment phase will focus on the identification of existing problems in performing daily activities and on risk factors for developing disability (i.e. polypharmacy, mobility, lack of social and productive activities, cognitive impairments). The PN and GP will determine if additional assessment is needed by the GP, PT, OT or other inpatient or outpatient specialists. At the end of the assessment phase the PN and GP will develop an intermediate action plan (step 3). Alternatively in cases of complex problems, the interdisciplinary team (i.e. PN, GP, PT and OT) will meet to formulate a shared action plan. Consequently, a meeting between the PN and the frail older person (and informal caregiver) will take place to define a final action plan, including goals, strategies and actions (step 4). The action plan will be tailored to the specific needs and wishes of the frail older person and will be related to a flexible toolbox of interventions which will be conducted by the interdisciplinary team (step 5). The toolbox can be supplemented with other interventions delivered by inpatient and outpatient specialists. The intervention protocol provides guidelines for referral to other disciplines. During execution and after finishing the components of the toolbox, the PN will evaluate, with the frail older person (and the informal caregiver), the achievement of goals, the implementation of strategies into daily life and the need for further support (step 6) (Daniëls et al.: Development of an intervention for community-dwelling frail elderly, submitted).

#### Training of health professionals

In the 3-month period before the start of the intervention, health professionals (PNs, GPs, PTs and OTs) received relevant training sessions with regard to the intervention protocol. Several meetings about the aspects and basic principles of the intervention protocol took place (i.e. the screening procedure, self-management principles, client centeredness, motivational interviewing, interdisciplinary collaboration, assessment tools, parts of the toolbox and referrals). Before the start of the study, health professionals had the opportunity to gain experience with the intervention protocol under supervision by the project team in small samples of frail older people who were not included in the study.

#### Control group

Frail older people in the control group were also selected by means of the screening questionnaire (GFI score ≥ 5). They will receive care as usual, and will be able to use or apply for all available services for older people as before.

### Outcome

#### Primary outcome measure

The primary outcome measure is disability. The Groningen Activity Restriction Scale (GARS) is an easy-to-administer, comprehensive, reliable, hierarchical and valid measure for assessing disability in the domains of activities of daily living (ADL), instrumental activities of daily living (IADL) and mobility in older people [34].

#### Secondary outcome measures

Secondary outcomes are depressive symptomatology (depression subscale Hospital Anxiety and Depression Scale) [35], social support interactions (Social Support List - Interaction version) [36], fear of falling (Short Falls Efficacy Scale - International) [37] and social participation (Maastricht Social Participation Profile) [38]. Feelings of loneliness will be assessed by the question: "During the past 4 weeks, how often did you feel lonely?" [19]. The frequency of falls will be assessed by the question: "How often did you fall during the past 6 months/12 months" [19]. Mortality, healthcare utilization and related costs will be continuously registered during 24 months.

#### Additional variables

Several additional variables will be assessed to provide insight into population characteristics and to interpret the outcomes of the study. Mental, physical, social, economic and behavioural factors may affect our primary outcome of disability [39]. Socio-demographic data (i.e. age, gender, marital status, living situation, educational level) will be gathered at baseline from the GFI and a standardized data set: the Minimal DataSet (MDS) (see Additional file 1: Minimal DataSet - care receiver). Other variables to be assessed at baseline and at 6, 12 and 24 month follow-up are cognitive impairment [40] and vision and hearing capacity [41].

We assume two variables to be potential effect modifiers. First, the frailty status at baseline (GFI score) as it predicts disability [5]. Second, feelings of competence and control, as they are crucial for self management and coping [42], which are important underlying mechanisms of the proposed intervention. Feelings of competence and control will be assessed at baseline and at 6, 12 and 24 month follow-up using the Mastery scale [43]. The impact of potential effect modifiers on disability will be studied in subgroup analyses.

Table 1 provides an overview of the instruments used to measure primary and secondary outcomes, and the additional variables.

**Table 1:** Primary, secondary and additional outcome measures

Variables	Instrument	No. of items	Range*	B	FU1	FU2	FU3
*Primary outcome measure*							

Disability	GARS [34]	18	18-72	TI	TI	TI	TI

*Secondary outcome measures*							

Cognitive impairment	TICS [40]	11	0-41	TI	TI	TI	TI
Symptoms of depression	HADS [35]	7	0-21	TI	TI	TI	TI
Social participation	MSSP [38]	10	0-90	TI	TI	TI	TI
Social support interactions	SSL12-I [36]	12	12-48	PQ	PQ	PQ	PQ
Fear of falling	Short FES-I [37]	7	7-28	TI	TI	TI	TI
No. of falls in the previous 6 months [19]	N/A	1	N/A	TI	TI	TI	TI
Consultation with physician due to fall	N/A	1	N/A	TI	TI	TI	TI
Feelings of loneliness [19]	N/A	1	N/A	PQ	PQ	PQ	PQ
Mortality	N/A	N/A	N/A	R	R	R	R

*Additional measures*							

Vision/hearing capacity	OECD-long-term disability indicator [41]	4	4-16	TI	TI	TI	TI
Mastery	Mastery scale [43]	7	7-35	TI	TI	TI	TI
Healthcare utilization	N/A	N/A	N/A	R	R	R	R

*The underlined scores indicate the most favourable scores; N/A = not applicable B = baseline; FU1 = 6-month follow-up; FU2 = 12-month follow-up; FU3 = 24-month follow-up; TI = telephone interview, PQ = postal questionnaire; R = registries of health insurance/GP/hospital

The proposed study will be embedded in the Dutch National Care for the Elderly Programme. This implies that the MDS has to be applied. The MDS for the care receiver provides global data on: age, gender, marital status, ethnicity, living arrangements, socio-economic status, level of education, health perception, multimorbidity, daily functioning in ADL, mental well-being, cognitive functioning, social functioning, quality of life and use of healthcare services. Data about the impact of the intervention on informal caregivers (perceived burden and health-related quality of life) will be gathered by the MDS for informal caregivers (see Additional file 2: Minimal DataSet - informal caregiver).

Data will be collected by a combination of two methods, telephone interviews (TI) and postal questionnaires (PQ), which have been proven to be feasible and efficient in previous research [18,19]. First, a second postal questionnaire after the screening questionnaire will be sent to frail older people to gather baseline data. Two weeks later a telephone interview will take place in order to gather additional information. These interviews will be conducted by independent interviewers of the Centre for Data and Information Management of Maastricht University (MEMIC), who will be blinded to the treatment assignment. The same procedure (postal questionnaire and telephone interview) will be repeated after 6, 12 and 24 months after baseline to gather follow-up data. In addition, data on healthcare utilization and related costs will be continuously gathered from health insurance registries and registries of the GPs and hospitals in the region. The central informal caregivers of the frail older people will receive a questionnaire at baseline and after 6, 12 and 24 months.

### Process evaluation

The process evaluation aims to improve further implementation of the intervention and to validate the results of the study. A systematic approach will be used for this evaluation, involving key elements such as reach, dose delivered, dose received (exposure and satisfaction) and barriers [44-46]. Table 2 gives a description of these elements.

**Table 2:** Elements of the process evaluation and data collection methods

Component and definition	Outcome variables	Measurement
**Reach** Proportion of the intended target population that participated in the intervention	Number of older people that refused, dropped-out or completed the intervention Reasons for refusal/drop-out (before start and during the intervention)	Diary

**Dose delivered (completeness)** Amount of delivered intervention	Assessments Referrals to other disciplines Interventions/toolbox parts Evaluation/follow-up	Diary

**Dose received (exposure)** Extent of active engagement in and receptiveness to the intervention by older people	Opinion about older peoples' ability to understand and implement principles of the intervention Adherence to commitments made by older people Intention of patients to implement the intervention	Questionnaire/interviews older people Questionnaire/interviews healthcare professionals

**Dose received (satisfaction)** Satisfaction of older people and healthcare professionals with the intervention	Overall opinion of older people Experienced benefits, burden, usefulness by older people Overall opinion of healthcare professionals	Questionnaire/interviews older people Questionnaire/interviews healthcare professionals

**Barriers** The extent to which problems were encountered while applying the intervention	Barriers in applying the intervention	Questionnaire/interviews healthcare professionals

**Figure 3:**
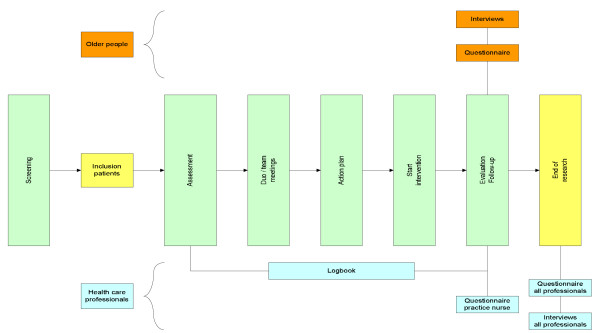
**Process evaluation plan**. The file contains an overview of the process evaluation plan.

Both quantitative and qualitative information will be collected from participants and healthcare professionals. Participants will evaluate the intervention by means of a self-administered questionnaire, directly after their last intervention contact. In addition, semi-structured interviews will be conducted among a random sample of participants to evaluate their experiences with the intervention. Questionnaires and semi-structured interviews will also be used among healthcare professionals to evaluate the intervention. In addition, they will be asked to register the treatments delivered (including the time spent on them) and reasons given for refusal and dropout throughout the intervention period. Figure 3 shows the data to be collected for the process evaluation during the study period.

### Economic evaluation

A cost-effectiveness analysis will be carried out in which costs will be considered from a societal perspective, which implies that all costs and effects are taken into account. The economic evaluation will be a combination of a cost-effectiveness and cost-utility analysis. The primary clinical outcome for the cost-effectiveness analysis is disability, which will be measured at baseline and at 6, 12 and 24 months follow-up by means of the GARS [34]. Within the cost-utility analysis, the primary outcome is generic health-related quality of life (QALY). Therefore utilities will be measured by means of the standard Dutch version of the EuroQol (EQ-6D) [47,48] at baseline and at 6, 12 and 24 months follow-up. Subsequently, a direct value for every state of health will be generated using the Dolan tariff [49,50], which involves an algorithm for interpolating EuroQol results to population utilities.

This study will assess intervention costs, healthcare costs and patient/family costs. Intervention costs relate to the delivery of the intervention, for example costs related to the screening procedure, time spend on intervention by healthcare professionals, travel expenses of professionals and costs for training activities. Data will be registered prospectively by the researchers. Healthcare costs relate to hospital visits (inpatient and outpatient treatment), GP consultations, visits to paramedics, prescribed medication and (nursing) home care. Data of healthcare utilization will be assessed by means of registries of health insurance agencies, GP practices and hospitals. In addition, questions about healthcare utilization are included in the postal questionnaire and the telephone interview. Patient and family costs relate to costs that are made by the patients or informal caregivers themselves and include, for example, travel expenses (based on the mean distance to and from healthcare professionals) and informal care (based on hours delivered). These data would preferably be assessed by means of a cost diary [51]. However, in the present study a diary was considered to be too burdensome for frail older people (and their informal caregivers).

In order to calculate costs, volumes of resource utilization will be multiplied by the cost price of that unit. Cost prices will be obtained from the Dutch guidelines for cost analysis in health care research [52,53]. Where such guidelines are not available for a specific category, real costs or tariffs will be used to estimate costs.

Differences in costs and effects will be presented in incremental cost-effectiveness ratios (ICERs). The ICERs represent the differences in mean costs between the intervention and usual care group in the numerator and the difference in mean effects in the denominator [54]. Sensitivity analysis will be done to assess the robustness of the assumptions we made.

### Sample size

The population sample size is based on the primary outcome measure of disability. Based on a previous study [19], we expect to demonstrate a difference in disability between the mean change score of the intervention and the control group of at least 2.0 points on the Groningen Activity Restriction Scale (GARS) [34] (which is equivalent to an effect size of 0.44 with SD 4.5). Based on a power of 80% and an alpha of 0.05 (two-sided testing), this leads to a minimal sample size of n = 80 per group (160 in total). Based on an expected drop-out rate of 30%, the required sample size would be n = 104 per group (n = 208 in total). However, the cluster randomized design of this study has consequences for the sample size and power. Scores of individuals within a cluster are assumed to be correlated in contrast to those of individuals between clusters. A within-cluster correlation leads to a greater homogeneity of individuals within a cluster, which increases the standard error of the estimate of the treatment effect. This may result in a loss of power for detecting differences between the intervention and control group. Therefore, an Intra Cluster Correlation (ICC) coefficient is needed to determine a corrected sample size [55]. In earlier intervention trials among GPs, ICC values between 0.03 and 0.06 were used [56-58]. In the present study an ICC value of 0.05 was estimated, resulting in a design effect of 1.7273. Based on the expected drop-out rate of 30%, the required sample size is n = 180 per group (n = 360 in total, an average of n = 30 per GP practice).

According to Puts and colleagues [59], the prevalence of frailty in Dutch older people aged 55 to 85 years old varies from 12% to 21%. We have conservatively estimated that 15% of the screened population will fulfil all criteria. A response rate on the postal screening of at least 65% was expected among older people [19,60]. On average, 300 screening questionnaires per GP practice (3,498 in total) were sent to older people to obtain a sufficient number of frail older people for the trial (n = 360). In addition, (central) informal caregivers will be included as well. It is expected that a central informal caregiver will be identified for 80% of frail older people.

### Statistical analysis

Descriptive techniques will be used to describe the study groups. Baseline variables will be compared to detect differences between the intervention and control groups at the start of the study. Data of the effect evaluation will be analysed according to the intention-to-treat principle. Analysis of primary and secondary endpoints will be performed using relevant univariate, multivariate and multilevel techniques including mixed-effects regression models. A subgroup analysis will be performed for frailty status and feelings of competence and control (mastery), as these variables are assumed to be potential effect modifiers. The software package SPSS for Windows, version 17.0., will be used for all statistical analyses. The level of statistical significance will be set at 0.05 (two-tailed).

Data on the process and economic evaluation will be analysed and presented using descriptive techniques and appropriate statistical testing. Data gathered from interviews (process evaluation) will be analysed using descriptive techniques.

## Discussion

Out of 52 GP practices, 24 practices have applied for taking part in the proposed study. This indicates a substantial interest of GPs in innovations regarding care for frail older people. Twelve of them were randomly selected to take part in the current study. A random selection of their community-dwelling older patients (≥ 70 years) was screened for frailty. For practical reasons the screening procedure was distributed across two cycles between December 2009 and April 2010. A total of 3,498 older people received the screening questionnaire. The response rate was 79.7%. According to the GFI scores 29.3% of the respondents can be considered as frail (GFI score ≥ 5). Nearly half of them (48.1%) were willing to participate. Sending out a postal questionnaire including the GFI [22] turned out to be a feasible and inexpensive method of identifying frail older people. Adding a letter from their general practitioners to the information leaflet may have contributed to the high response rate.

The proposed study has some potential limitations. First, the participating GPs are very interested in innovations in the care of older people otherwise they would not have applied to take part in the study. Consequently, the GPs allocated to the control group may take initiatives to improve the care for the older people in this group during the study period. The researchers will carefully monitor the activities of GPs regarding the potential improvement of care of these older people. Second, implementation of the intervention protocol is a point of concern. Based on a combination of elements such as interdisciplinary decision-making and collaboration (i.e. team meetings), self-management (taking principles of client-centredness, behavioural change and motivational interviewing into account), and an extensive toolbox of interventions, the intervention is very complex. Educating and guiding teams in implementing the programme are therefore important.

### Progress of study

The baseline measurements started in January 2010. In February 2010 the first frail older people were approached by the PN for a comprehensive assessment. Data on the effect evaluation will be available in 2012. Data for the process evaluation and economic evaluation will be gathered between 2010 and 2012.

## Abbreviations

GFI: Groningen Frailty Indicator; CGA: Comprehensive Geriatric Assessment; GP: general practitioner; PN: practice nurse; PT: physiotherapist; OT: occupational therapist; GARS: Groningen Activity Restriction Scale; ADL: Activities of Daily Living; IADL: Instrumental Activities of Daily Living; MDS: Minimal DataSet; TICS: Telephone Interview Cognitive Status; HADS: Hospital Anxiety and Depression Scale; SSL 12-I: Social Support List; FES: Falls Efficacy Scale; OECD: Organization for Economic Cooperation and Development; N/A: not applicable; B: baseline; FU: follow-up; TI: telephone interviews; PQ: postal questionnaires; MEMIC: Centre for Data and Information Management of Maastricht University; QALY: generic health-related quality of life; EQ-6D: standardized instrument for use as a measure of health outcome; ICERs: incremental cost-effectiveness ratios; SD: standard deviation; N: sample size; ICC: Intra Cluster Correlation; SPSS: Statistical Program for the Social Sciences.

## Competing interests

The authors declare that they have no competing interests.

## Authors' contributions

SM, EvR, LdW and RK were responsible for the research questions. All authors contributed to drafting of the study protocol. SM made the first draft of this paper. The other authors commented on it and approved the final version. MH was consulted for the economic evaluation.

## Pre-publication history

The pre-publication history for this paper can be accessed here:

http://www.biomedcentral.com/1471-2458/10/511/prepub

## Supplementary Material

Additional file 1 **Minimal DataSet (MDS) - care receiver**. The file contains an overview of all items of the MDS for the care receiver. Click here for file

Additional file 2 **Minimal DataSet (MDS) - informal caregiver**. The file contains an overview of all items of the MDS for the informal caregiver. Click here for file
