# Supplementary material for: The reduction of disability in community-dwelling frail older people: design of a two-arm cluster randomized controlled trial
Source: BMC Public Health. 2010 Aug 23;10:511. doi: 10.1186/1471-2458-10-511 (PMC2936429; doi:10.1186/1471-2458-10-511)
Supplement: Additional file 1 — Minimal DataSet (MDS) - care receiver. The file contains an overview of all items of the MDS for the care receiver. [file 1471-2458-10-511-S1.DOC]

**Additional file 1: Minimal DataSet – care receiver**

| **Domein** | **Operationalisation** | **No. of items** |
| --- | --- | --- |
| *Minimal DataSet - care receiver* | | |
| Age | Date of birth | 1 |
| Gender | - Female - Male | 1 |
| Socio-economic status / geographical distance to caregiver | Numbers of postal code home address | 1 |
| Ethnicity | Country of birth care receiver  Country of birth father care receiver  Country of birth mother care receiver  Format according to GGD monitor [1] | 3 |
| Educational level | Format according to Verhage 1966 [2] | 1 |
| Marital state | - Married - Divorced - Widow/ widower/ partner deceased - Unmarried - Living together, unmarried | 1 |
| Living situation | - Independent, alone - Independent, with others (e.g. partner, offspring) - Home for the aged/ care home since… - Nursing home since… | 2 |
| Perceived health | Questions 1, 2 RAND-36 [3] | 2 |
| Utility care receiver | EQ-6D [4] | 7 |
| Multimorbidity | Simplified version of question on morbidity from GGD Monitor [1] | 1 |
| Functioning in (Instrumental) Activities of Daily Living | Katz-15 [5] | 15 |
| Psychological well-being | Subscale Mental Well-being RAND-36 [3] | 6 |
| Social functioning | Question 10 RAND-36 [3] | 1 |
| Perceived Quality of Life perceptions | “In general, would you say your quality of life is?”  Phrasing according to question 1 RAND-36 [3] | 1 |
|  | “How would you rate your life at this moment?”  Variation on Cantril’s Self Anchoring Ladder [6] | 1 |
|  | “Compared to one year ago, how would you rate your quality of life in general now?”  Conform question 1 RAND-36 [3] | 1 |
| Health care use | Hospital stays | 2 |
|  | Out-of-office GP care | 2 |
|  | Home care | 1 |
|  | Temporary stay in care home | 1 |
|  | Temporary stay in nursing home | 1 |
|  | Day care | 1 |
|  | Day treatment | 1 |

**References**

1. **Lokale en nationale monitor gezondheid** [<http://www.monitorgezondheid.nl/home.xml>]

2. Verhage F: *Intelligence and age: Study with Dutch people from age 12 to 77*. Assen: Van Gorkum; 1964.

3. Van der Zee K, Sanderman R: **Het meten van de algemene gezondheidstoestand met de Rand-36: een handleiding** 2002.

4. Krabbe PFM, Stouthard MEA, Essink-Bot M-L, Bonsel GJ: The Effect of Adding a Cognitive Dimension to the EuroQol Multiattribute Health-Status Classification System - The Nottingham Health Profile, the MOS 36-Item Short-Form Health Survey, the COOP/WONCA charts, and the EuroQol instrument. J Clin Epidemiol 1999, 52(4):293-301.

5. Weinberger M, Samsa GP, Schmader K, Greenberg SM, Carr DB, Wildman DS: **Comparing proxy and patients' perceptions of patients' functional status: results from an outpatient geriatric clinic**. *J Am Geriatr Soc* 1992, **40**(6):585-588.

6. Cantril H: *The pattern of human concerns*. New Brunswick: Rutgers University Press; 1965.
