# Supplementary material for: The reduction of disability in community-dwelling frail older people: design of a two-arm cluster randomized controlled trial
Source: BMC Public Health. 2010 Aug 23;10:511. doi: 10.1186/1471-2458-10-511 (PMC2936429; doi:10.1186/1471-2458-10-511)
Supplement: Additional file 2 — Minimal DataSet (MDS) - informal caregiver. The file contains an overview of all items of the MDS for the informal caregiver. [file 1471-2458-10-511-S2.DOC]

**Additional file 2: Minimal DataSet – informal caregiver**

| *Minimal DataSet - informal caregiver* | | |
| --- | --- | --- |
| Age | Date of birth | 1 |
| Gender | - Female - Male | 1 |
| Socio-economic status caregiver/ geographical distance to care receiver | Numbers of postal code home address | 1 |
| Age care receiver | Date of birth | 1 |
| Gender care receiver | - Female - Male | 1 |
| Socio-economic status care receiver / geographical distance to caregiver | Numbers of postal code home address | 1 |
| Relationship to care receiver | - Spouse / partner - Sister / brother (in law) - Daughter / son (in law) - Other | 1 |
| Living together with care receiver | - Yes - No | 1 |
| Perceived health | Question 1, 2 RAND-36 [1] | 2 |
| Number of hours of informal care | Short version Erasmus iBMG measuring instrument “objective burden informal care” [2] |  |
| Perceived caregiving burden | Self-Rated Burden VAS and Carer-QoL [3] | 10 |
| Perceived quality of life | “In general, would you say your quality of life is?”  Phrasing according to question 1 RAND-36 [1] | 1 |
|  | “How would you rate your life at this moment?”  Variation on Cantril’s Self Anchoring Ladder [4] | 1 |
|  | “Compared to one year ago, how would you rate your quality of life in general now?”  Conform question 1 RAND-36 [1] |  |

**References**

1. Van der Zee K, Sanderman R: **Het meten van de algemene gezondheidstoestand met de Rand-36: een handleiding** 2002.

2. Van den Berg B, Spauwen P: **Measurement of informal care: an empirical study into the valid measurement of time spent on informal caregiving**. *Health Econ* 2006, **15**(5):447-460.

3. Brouwer WB, van Exel NJ, van Gorp B, Redekop WK: **The CarerQol instrument: a new instrument to measure care-related quality of life of informal caregivers for use in economic evaluations**. *Qual Life Res* 2006, **15**(6):1005-1021.

4. Cantril H: *The pattern of human concerns*. New Brunswick: Rutgers University Press; 1965.
